# ^134^Cs Uptake and Growth at Various Cs^+^ and K^+^ Levels in *Arabidopsis* *AtKUP7* Mutants

**DOI:** 10.3390/plants9111525

**Published:** 2020-11-09

**Authors:** Marek Šustr, Tereza Doksanská, Barbora Doležalová, Aleš Soukup, Edita Tylová

**Affiliations:** 1 Department of Experimental Plant Biology, Faculty of Science, Charles University, Viničná 5, 128 44 Prague 2, Czech Republic; sustrm@natur.cuni.cz (M.Š.); b.cabelkova@seznam.cz (B.D.); asoukup@natur.cuni.cz (A.S.); 2 National Radiation Protection Institute, Bartoškova 28, 140 00 Prague 4, Czech Republic; tereza.doksanska@suro.cz

**Keywords:** caesium, root growth, potassium, KT/HAK/KUP, AtKUP7

## Abstract

Radiocaesium is a pollutant with a high risk for the environment, agricultural production, and human health. It is mobile in ecosystems and can be taken up by plants via potassium transporters. In this study, we focused on the role of potassium transporter AtKUP7 of the KT/HAK/KUP family in Cs^+^ and K^+^ uptake by plants and in plant tolerance to caesium toxicity. We detected that *Arabidopsis* *kup7* mutant accumulates significantly lower amounts of ^134^Cs in the root (86%) and in the shoot (69%) compared to the wild-type. On the other hand ability of the mutant to grow on media with toxic (100 and 200 µM) concentrations of Cs^+^ was not changed; moreover its growth was not impaired on low K^+^. We further investigated another mutant line in *AtKUP7* and found that the growth phenotype of the *kup7* mutants in K^+^ deficient conditions is much milder than previously published. Also, their accumulation of K^+^ in shoots is hindered only by severe potassium shortage.

## 1. Introduction

Caesium is very rare in natural soils as it constitutes only about 3 ppm of soil mass. However, soils can be enriched in anthropogenic radioactive caesium due to nuclear weapons testing and nuclear power plant accidents. The most prominent events concerning the spread of radiocaesium in northern hemisphere were tests of nuclear weapons and the Chernobyl disaster in April 1986 [1]. More recently, large amounts of caesium were released into terrestrial and marine ecosystems when the Fukushima Daiichi nuclear power plant was damaged by a tsunami in 2011 [1,2,3].

Common radionuclides of caesium are ^137^Cs and ^134^Cs [4]. With relatively long half-lives of 30 and 2 years respectively, these radionuclides persist in the soil and are introduced into the food chain through plant uptake. Indeed, caesium is readily transported into plants as a chemical analogue of potassium [4]. This led researchers to study soil and genetic factors influencing caesium uptake to reveal risks of crop production in contaminated soils as well as the potential to remediate contaminated soil via plants.

So far, numerous studies founded that a limited supply of potassium from soils or cultivation media increased caesium uptake in various plant species, including wheat [5], spinach [6], and beans [7]. These studies highlight candidate proteins for caesium transport, which are components of a high-affinity potassium uptake system [8], directed towards caesium transport during potassium scarcity [9]. Primarily these are transporters from the KT/HAK/KUP (K^+^ transport/High-affinity K^+^/K^+^ uptake) family. In *Arabidopsis thaliana*, the single transporter upregulated in response to low potassium availability is AtHAK5 and its involvement in caesium uptake has been reported [10,11]. Some other KT/HAK/KUP transporters were also indicated to affect caesium accumulation in *A. thaliana* [12]. In tomato SlHAK5, an orthologue of AtHAK5, seems to mediate most of the caesium influx in conditions of potassium scarcity [13]. In rice, mutagenesis revealed OsHAK1, a close relative of AtHAK5, as the transporter responsible for large part of caesium influx [14]. A quantitative genetic approach in *A. thaliana* indicated two other transporters from different families that correlate with enhanced caesium sequestration: AtCHX16 (Cation/H^+^ exchanger 16), and AtCNGC1, (Cyclic nucleotide-gated channel 1) [15]. These transport proteins have not yet been functionally characterised. Reduced caesium accumulation was also shown in a SNARE (SNAP receptor) protein *sec22* (Secretion deficient 22) mutant. In *A. thaliana*, accumulation of caesium was decreased in *Atsec22* mutant plants and yeast *Scsec22* mutants had impaired transport of caesium into the vacuole [16].

When analysing the risks of radiocaesium soil-plant translocation for food production and human health, root-shoot transport and allocation to edible parts of the plant are of highest concern. Consumption of plants with high content of radiocaesium leads to internal radiation with more severe consequences. Among other examples, it was shown in rice, that a higher amount of caesium is transported into the shoot during fruit ripening [17]. Also, brown rice had higher Cs^+^/K^+^ ratio than the rest of the shoot [18]. In summary, Cs^+^ accumulation is generally higher in fast-growing species, although it differs among taxonomic groups [19], and cultivars [20,21,22]. High Cs^+^ accumulation occurs in species of Chenopodiaceae, Amaranthaceae, and Brassicaceae families [19,23].

Transporters involved in K^+^ xylem loading were identified and are good candidates for proteins mediating radiocaesium translocation from root system to shoot. In *A. thaliana*, shaker-type channel SKOR (Stellar K^+^ outward rectifying channel) participates in xylem potassium loading [24], but its involvement in caesium transport is not known. Also, non-canonical potassium transporters are involved in potassium and caesium partitioning. NRT1.5 (Nitrate transporter 1.5) is involved in potassium xylem loading, with unknown discrimination between K^+^ and Cs^+^[25], while ZIFL2 (Zinc-induced facilitator-like transporter 2) is specific for caesium partitioning. When *ZIFL2* is mutated, caesium is accumulated more in the *A. thaliana* shoot [26]. Recently, transporters that accumulate potassium in xylem parenchyma were identified. KT/HAK/KUP transporter OsHAK5 participates in both potassium uptake and xylem loading in rice [27]. In *A. thaliana* another KT/HAK/KUP transporter, AtKUP7, is also involved in potassium uptake as well as accumulation in xylem parenchyma and affects potassium loading into the xylem [28]. It is, therefore, a highly probable candidate for participating in the root-shoot translocation of radiocaesium in *A. thaliana*.

In this study, we focused on AtKUP7 transporter and its possible involvement in uptake and root-shoot transport of ^134^Cs^+^ and K^+^. We examined T-DNA insertional mutant of *AtKUP7* (*kup7-1*) for its capacity to take up ^134^Cs^+^ and K^+^ and translocate these ions into the shoot. The sensitivity of *kup7-1* mutant plants to Cs^+^ toxicity and K^+^ scarcity was also analysed.

We found, that *kup7-1* accumulates less radiocaesium in both roots and shoot. In addition, we observed a much milder growth phenotype in low K^+^ conditions than previously published for another mutant line (marked *kup7-2* in this study). Therefore, we compared these two mutant lines in subsequent cultivation experiments. We found that low K^+^ phenotypes of *Atkup7* mutants are generally milder than previously published.

## 2. Results

### 2.1. Plant Material

After purchase, both mutant lines were genotyped by PCR and homozygous plants were backcrossed with Col-8. Homozygotes and respective wild-types were selected in the F2 generation with PCR genotyping. T-DNA insertion was confirmed in the annotated position by sequencing. In *kup7-1*, the presence of transcript was evaluated by qualitative RT-PCR. Transcript upstream of the insertion was present in the homozygotes, while the transcript in the vicinity of the insertion was not detectable (Supplementary Figure S1).

### 2.2. Short-term Uptake of ^134^Cs^+^

**Figure 1:**
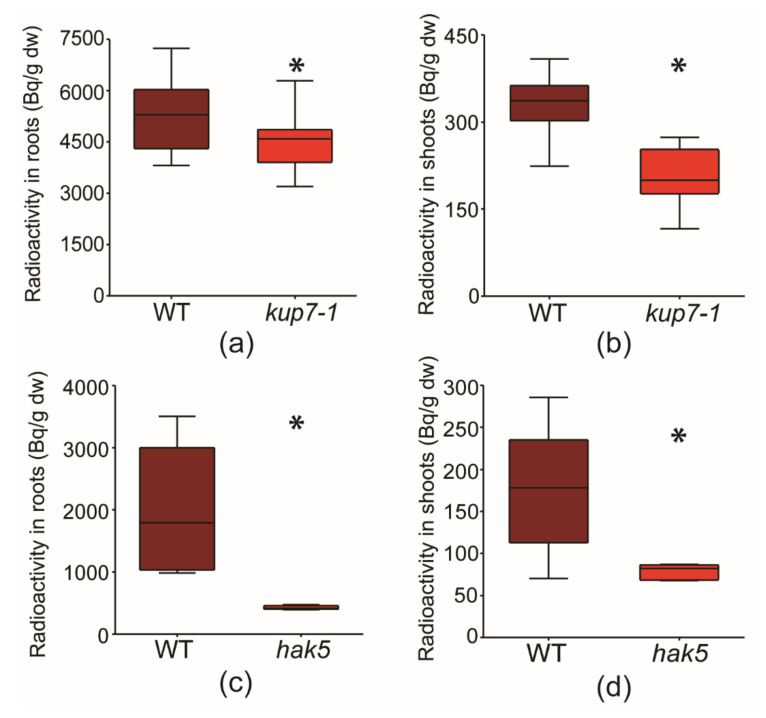
The radioactivity (Bq.g^−1^ of dry weight) in plant parts after 24h incubation in solution containing 1800 Bq.l^−1 134^CsCl and 0.5 mM CaCl_2_. Radioactivity in (**a**) roots and (**b**) shoots of *kup7-1* plants and respective wild types and in (**c**) roots and (**d**) shoots of *hak5* and respective wild types. * indicates significant differences between genotypes (Aspin–Welch Unequal-variance T-test, *p* ≤ 0.05, n = 6–8).

After 24 h exposure to ^134^CsCl, *kup7-1* plants contained significantly less radioisotope than the wild-type plants in both roots and shoots (Figure 1a,b). ^134^Cs activity in *kup7-1* roots was 86% of the wild type, while it was 69% of the wild type in the shoot. This suggests that both root uptake and root-shoot transport of ^134^Cs were hindered by the *KUP7* mutation. Both mutant and wild-type plants had same growth rate on the basis of dry weights at the time of ^134^Cs incubation experiment (after 3 weeks of cultivation in K^+^ replete medium + 1 week of K^+^ starvation; see Supplementary Figure S2).

We also evaluated the sensitivity and reliability of our ^134^Cs incubation methods using published *hak5* mutant line (SALK_005604C). In the same experimental setup, the *hak5* mutant exhibited significantly lower ^134^Cs uptake into its roots (21%) and shoot (45%) compared to wild-type (Figure 1c,d), which is in agreement with its function in caesium uptake [11].

### 2.3. Plant Susceptibility to ^133^Cs^+^ Toxicity

**Figure 2:**
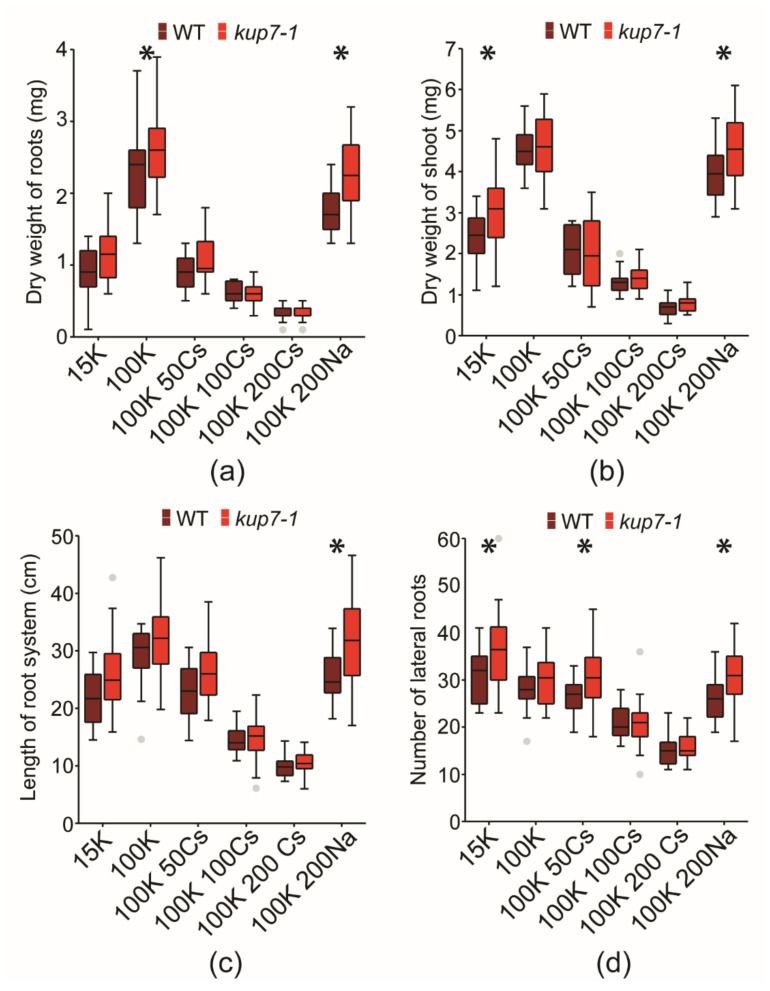
Growth metrics of wild type and *kup7-1* plants after 16 days (**a**,**b**) or 10 days (**c**,**d**) of cultivation on various K^+^ and Cs^+^ levels. (**a**) Dry weight of roots, (**b**) dry weight of shoots, (**c**) total length of root system, (**d**) number of lateral roots. * indicates significant differences between genotypes within treatments (Aspin–Welch Unequal-variance T-test, *p* ≤ 0.05, n = 24).

**Figure 3:**
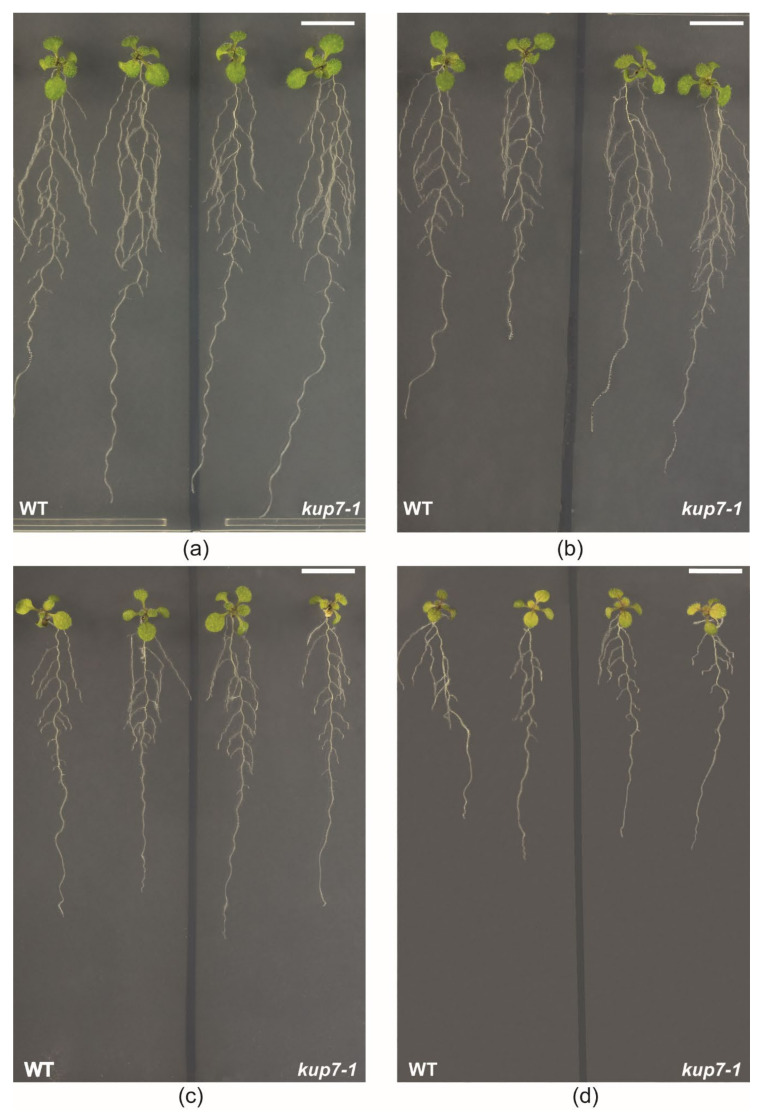
Phenotype of wild type and *kup7-1* plants on (**a**) 100 µM K^+^, (**b**) 50 µM Cs^+^ and 100 µM K^+^, (**c**) 100 µM Cs^+^ and 100 µM K^+^ and (**d**) 200 µM Cs^+^ and 100 µM K^+^, scale bars 1 cm.

The significantly lower uptake of ^134^Cs by *kup7-1* mutants might suggest reduced long-term accumulation of caesium in *kup7-1.* Since caesium can be toxic to plants and its toxicity depends on potassium supply [29], we tested the growth of *kup7-1* plants in different caesium concentrations and in low potassium availability. In this experiment, we used media with three concentrations of stable caesium isotope (50, 100 and 200 µM ^133^CsCl) supplied with 100 µM of K^+^, which enabled reasonable growth of the plants. Growth of plants was gradually inhibited by rising concentrations of caesium. Dry weights of roots and shoots, total length of root system, and number of lateral roots were significantly lower in these treatments compared to plants exposed to 100 µM K^+^ only (GLM ANOVA, *p* ≤ 0.05; Figure 2a–d and Figure 3). We also included the treatment with 100 µM K^+^ + 200 µM NaCl to our experimental design. The growth of Na^+^ treated plants was the same as in Cs^+^ free treatment (GLM ANOVA, *p* ≤ 0.05; Figure 3a–d), which proved that the observed growth inhibition was caesium-specific.

The comparison of genotypes showed, that overall growth of *kup7-1* mutants was slightly better than wild type. This phenotype was statistically significant for root and shoot dry weights as well as for total lengths of root systems (GLM ANOVA, *p* ≤ 0.05). On the other hand, the genotypes within individual treatments (Aspin–Welch Unequal-variance T-test, *p* ≤ 0.05) showed significant differences between *kup7-1* and wild-type plants—mostly in Cs-free treatments, while differences were never found at 100 and 200 µM Cs^+^ levels (Figure 2a–d).

### 2.4. Response to K^+^ Shortage

To test the response to severe potassium starvation, we used a treatment with 15 µM K^+^. Plants on this media grew significantly less than on the 100 µM K^+^ media, although still better than those on media with addition of 100 and 200 µM Cs^+^ (GLM ANOVA, *p* ≤ 0.05; Figure 2a–c). The only exception to this trend was root branching. The depletion of K^+^ to 15 µM enhanced root system branching in comparison to 100 µM K^+^ (GLM ANOVA, *p* ≤ 0.05; Figure 2d).

Surprisingly, *kup7-1* plants grew slightly better than wild type. They exhibited higher shoot biomass and number of lateral roots after 16 and 10 days of K^+^ shortage respectively (Figure 2b,d). This growth trend did not correspond with data published for another *kup7* line (SAIL_105_G04) [28]. We, therefore, included this line (marked as *kup7-2*) and the media from previously mentioned study to compare low K^+^ responses of both lines. On both types of low K^+^ media, *kup7-1* plants showed slightly enhanced growth compared to wild type; they had significantly higher shoot biomass (Aspin–Welch Unequal-variance T-test, *p* ≤ 0.05; Supplementary Figure S3a–d). On the other hand, growth of *kup7-2* did not significantly differ from its wild type in any biometric parameter (Aspin–Welch Unequal-variance T-test, *p* ≤ 0.05; Supplementary Figure S3a–d).

### 2.5. Potassium Accumulation in Low K^+^ Conditions

**Figure 4:**
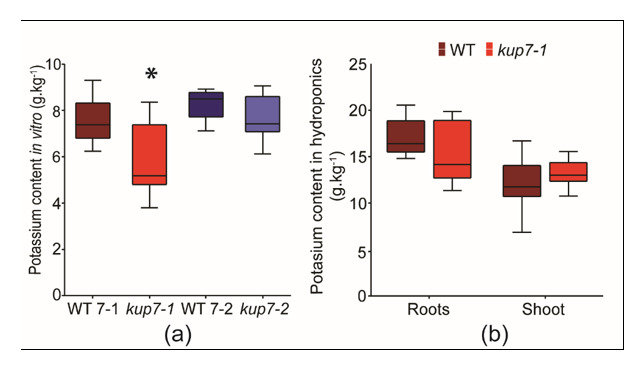
Potassium contents (g.kg^−1^ dry weight) of wild type and *kup7* plants. (**a**) Shoots of *kup7-1* and *kup7-2* supplied with 100 µM K^+^ in vitro for 16 days. (**b**) Roots and shoots of WT and *kup7-1* grown in hydroponics for three weeks in 4 mM K^+^ and one week in 15 µM K^+^.* indicates significant differences between genotypes within treatments (Aspin–Welch Unequal-variance T-test, *p* ≤ 0.05, n = 11).

The potassium content in plant biomass was analysed in plants grown for 16 days on agar plates supplied with 100 µM K^+^. In this experimental setup, *kup7-1* plants grew significantly better than other genotypes and had longer primary roots during the cultivation. *Kup7-2* showed no difference compared to wild type (Aspin–Welch Unequal-variance T-test, *p* ≤ 0.05; Supplementary Figure S4a). Shoots of *kup7-1* mutants contained significantly less potassium than their respective wild type, while reduction of potassium content in shoots of *kup7-2* was not significant (*p* = 0.07; Aspin–Welch Unequal-variance T-test; *p* ≤ 0.05, Figure 4a).

Potassium content was also analysed in 4-week-old *kup7-1* plants growing for 3 weeks in full potassium media (4 mM K^+^) and for 1 week in 15 µM K^+^ in hydroponics. At the end of this cultivation, overall growth of *kup7-1* was better than wild type. The difference was statistically significant for root biomass (Aspin–Welch Unequal-variance T-test, *p* ≤ 0.05; Supplementary Figure S4b). In terms of potassium accumulation, both shoot and root potassium contents did not differ significantly between wild type and mutant (Aspin–Welch Unequal-variance T-test, *p* ≤ 0.05; Figure 4b).

## 3. Discussion

### 3.1. Radiocaesium Accumulation and Cs^+^ Sensitivity of kup7-1 Mutant Plants

In case of any accidental release of caesium radioisotopes into the environment, three factors determine its impact on the food chain and agricultural production: ecosystem mobility of the radioisotope, soil-plant uptake, and root-shoot translocation. Soil characteristics affect the caesium mobility and soil-plant translocation, but species-specific differences in soil-shoot transfer factors seem to be determined mostly by root-shoot transport [30]. As summarized by Burger and Lichtscheidl [31], some species keep caesium in root system, while others (including *A. thaliana*) export substantial amounts of Cs^+^ to the shoot. Root uptake of caesium is mediated by some potassium transporters, e.g., AtHAK5, which is the critical component of high-affinity K^+^ uptake in low K^+^ conditions [10,11]. The mechanisms of caesium translocation to the shoot have gotten much less experimental attention.

In our study, we focused on AtKUP7 transporter, a very promising candidate for a protein involved in radiocaesium translocation to the shoot in *A. thaliana* because of its role in potassium loading into the xylem [28]. We have found that *Arabidopsis kup7-1* mutant plants display significantly lower root-shoot translocation of ^134^Cs compared to wild type plants in K-limiting conditions. Both short-term root uptake and shoot-ward transport of ^134^Cs^+^ were impaired in *kup7-1*, the latter to a greater extent (Figure 1a,b). These data are in agreement with the role of AtKUP7 in potassium transport to the shoot [28]. In contrast, no difference in long-term Cs^+^ accumulation between *A. thaliana kup7* and wild type seedlings were found after 7 days of cultivation in the study of [12]. This can, however, be explained by relatively high K^+^ availability used in their experiment (300 µM CsCl + 500 µM KCl), which might prevent significant involvement of AtKUP7 in K^+^ or Cs^+^ accumulation. The transport activity of AtKUP7 is important, especially in low-K conditions [28]. Moreover, *A. thaliana* translocates a larger proportion of caesium accumulated in roots to the shoot, particularly in K-sufficient conditions [32]. These currents might be mediated by SKOR [24] NRT 1.5 [25] or other unknown components, although their involvement is yet to be tested.

To verify our experimental setup for Cs^+^ uptake measurement, we included *A. thaliana hak5* mutant plants. The AtHAK5 transporter is well known as the key component for potassium uptake in K^+^ limiting conditions, and also being involved in Cs^+^ uptake by roots [10,11]. We successfully evaluated our approach as *Athak5* plants showed severely reduced ^134^Cs^+^ uptake, in agreement with previously published data [11]. In the K^+^ deficient conditions induced by our treatment, short-term root accumulation of ^134^Cs^+^ in *Athak5* plants was 21% compared to wild type. Under the same conditions, *kup7-1* plants root accumulation of ^134^Cs^+^ was 86% and the accumulation in shoot was 69% compared to wild type (Figure 1c,d).

Such comparisons between *Athak5* and *Atkup7-1* indicates that *AtHAK5* has an irreplaceable function in K^+^ limiting conditions [33], while *AtKUP7* is more redundant in K^+^ uptake and translocation to shoot. Alternatively, *AtKUP7* might be more selective for K^+^ over Cs^+^. The ability to transport Cs^+^ was proven by heterologous expression in yeast for other members of KT/HAK/KUP family, e.g., AtHAK5 [34] or AtKUP9 [35]. AtKUP7 needs to be tested in the same way to get final proof of its Cs^+^ transport ability. The role of AtKUP7 might also be indirect, via regulation of other transporters. A functional adenylate-cyclase domain was detected on N-terminal cytoplasmic domain of AtKUP7 [36] as well as AtKUP5 [37], and the role of this domain in regulation of K^+^ homeostasis was proposed [37]. All these possibilities need to be verified by future experiments.

We also tested whether the reduced short-term uptake and translocation of Cs^+^ improves the growth of *Atkup7-1* in the presence of Cs^+^ in the rhizosphere. Cs^+^ distribution in plant tissues is similar to K^+^ [38] and Cs^+^ toxicity correlates with the Cs/K ratio of the plant [29]. In agreement, enhanced tolerance to Cs^+^ toxicity was previously documented for *Athak5* plants and linked with their lower Cs^+^ uptake in low K^+^ conditions [11]. We used the stable isotope ^133^Cs in these experiments. The addition of 50–200 µM of Cs^+^ into the 100 µM K^+^ media-induced mild to severe Cs^+^ toxicity as was published for *A. thaliana* [11]. The response of *Atkup7-1* and wild-type plants to Cs^+^ did not differ significantly in the vast majority of growth parameters at any Cs^+^ level applied (Figure 2a–d). We can assume that the reduction in uptake of Cs^+^ by 14% and reduced shoot-ward transport by 31% in *kup7-1* is too small of a change to affect plant growth in a Cs-rich environment. It is also possible that higher Cs^+^ translocation to the shoot in wild-type plants is later compensated by transport systems that re-translocate Cs^+^ back into the root and environment. This may correspond with the data published by Adams, Miyazaki and Shin [12], who did not detect differences in long-term Cs^+^ accumulation between *kup7* and wild-type plants. As summarized by [8] and [39], the majority of Cs^+^ delivered to the shoot can be recirculated to the roots via the phloem. An example of a phloem re-translocation system might be the metal-stabilising protein NaKR1. Mutants in *AtNaKR1* accumulate various alkali ions (K^+^, Na^+^ and Rb^+^) in their shoots [40], but the role of NaKR1 in Cs^+^ phloem transport needs to be experimentally tested.

### 3.2. Low K^+^ Responses and K^+^ Accumulation in Arabidopsis kup7 Mutants

Growth retardation and reduced shoot K^+^ accumulation in *Atkup7* seedlings cultivated in low K^+^ conditions (100 µM K^+^) were previously shown as evidence indicating the involvement of AtKUP7 in xylem loading [28]. In agreement with this, we detected lower K^+^ contents in shoots of *Atkup7-1* plants in similar conditions (short-term in vitro cultivation on media with 100 µM K^+^) (Figure 4a). On the other hand, *Atkup7-1* plants grew slightly but often significantly better than wild-type plants in short-term in vitro cultivations with 15-100 µM K^+^ levels (Figure 2a–d). This phenotype was also observed in long-term hydroponic cultivation where severe low-K conditions (15 µM K^+^) were applied during the last week of four-week cultivation, after three weeks of growth in K-replete solution (4 mM K^+^). Despite this, we did not find any difference in shoot K^+^ contents between *Atkup7-1* and wild type plants in hydroponics (Figure 4b).

In plants pre-treated with a potassium-rich media for a long period of growth, the function of AtKUP7 might be redundant. AtKUP7 is expressed in most cell types in the root and actively transports K^+^ into the cells, thus contributes to K^+^ accumulation in xylem parenchyma cells [28]. Then K^+^ is released into xylem vessels by the shaker-like channel AtSKOR [24] and K^+^/H^+^ antiporter AtNRT1.5 [25]. AtKUP7 thus contributes to potassium uptake into the cells [28] but its involvement in root K^+^ uptake from rhizosphere is less significant in comparison to AtHAK5 and AtAKT1 (*Arabidopsis* K^+^ transport) [41]. In addition, disruption of KUP7 function in K^+^ uptake does not manifest in potassium-rich conditions [28], which might be similar to our plants cultivated in potassium replete conditions three weeks prior the period of K^+^ starvation.

Slightly better growth of *Atkup7-1* compared to wild-type seedling in low-K conditions is surprising as it does not correspond with the growth retardation of *Atkup7* mutants described by [28]. However, we used another mutant line (SALK_206158S*; Atkup7-1*), where the T-DNA insertion is located in the last exon of *AtKUP7*. By qualitative RT-PCR we detected that the transcript upstream of the insertion is present in homozygous plants and only the downstream part is disrupted (Supplementary Figure S1). Hence, we cannot exclude the presence of a partial protein with a missing part of the C-terminal domain. The study of [28] worked mostly with the mutant line SAIL_105_G04 (*Atkup7*-2). In this line, the insertion is located in the first exon of *AtKUP7*, the transcript is not present, and the transporter is disrupted completely [28]. Therefore, we included this line in our study to compare low-K growth phenotypes of both lines. In low-K conditions, seedlings of both lines tended to accumulate less K^+^ in the shoot (Figure 4a). However, they differed in growth parameters. *Atkup7-1* plants showed slightly enhanced growth, while *Atkup7-2* plants tended to be smaller compared to their respective wild type or did not differ (Supplementary Figure S3a–d). We also grew both lines on a medium resembling that from its original publication, which contained 1.25 mM NH_4_^+^. NH_4_^+^ may negatively affect plants that suffer from potassium shortage [42], and inhibits the expression of AtHAK5 [11]. Both lines grew slightly worse on the basis of shoot dry weight on this medium but maintained the differences in growth rates (Supplementary Figure S3a–d). In our hands, the growth phenotype of K^+^ deficient *Atkup7* plants was generally milder than previously published for *Atkup7-2.* However, the difference between *kup7-1* and *kup7-2* mutant lines exists independent of cultivation conditions.

We cannot exclude that, in the *Atkup7-1* line, a partial KUP7 protein can be formed. This protein might have a part of the cytosolic C-terminal domain disrupted by T-DNA insertion in *AtKUP7* gene, but the remaining part might be partially functional. This part includes the transmembrane domains of the transporter’s pore, as well as the AMP cyclase domain on N-terminus [36]. Little is known about the function of this domain. In *kup7-2* line, AtKUP7 protein is completely missing as Han, Wu, Wu and Wang [28] did not detect any *AtKUP7* partial transcript. We can speculate that the presence of an AMP cyclase domain or the gain of function conformation change in this domain might be the cause of enhanced growth in *kup7-1*. This interesting possibility needs to be tested.

## 4. Conclusions

This research and previous work show, that two distinct mutant lines in *AtKUP7* have decreased root-shoot transport of alkali ions (Cs^+^ and K^+^). This decrease is not uniformly translated into lower potassium content in plants’ shoot. Also, we did not observe any growth retardation in either mutants. Therefore, we conclude that the phenotype of *KUP7* mutation is much milder than previously thought and its function is probably redundant.

## 5. Materials and Methods

### 5.1. Plant Material

Homozygous mutant T-DNA insertion line for *AtKUP7* (At5g09400): *kup7-1* (SALK_206158S) of *Arabidopsis thaliana* (L.) Heynh. was used in this study. Additionally, another *kup7* line (SAIL_105_G04, marked *kup7-2* in the text) and insertion line for *AtHAK5* (At4g13420)—*hak5* (SALK_005604C) were used. Seeds of all the lines were obtained from publicly available collections (NASC, Nottingham Arabidopsis Stock Centre) and used in experiments. Lack of the wild-type allele (the presence of T-DNA insertion) was confirmed by standard genotyping PCR procedure using pre-designed genotyping primers from SALK Institute website [43]. Position of the insertion was confirmed by sequencing. Mutant plants were backcrossed into their genetic background (Col-8; N60000) and homozygous mutants and wild-type controls were selected in F2 generation by genotyping. RNA was isolated from *kup7-1* homozygotes using the NukleoSpin RNA Plant kit (Mecherey-Nagel; Duren, Germany) and the presence of transcript in these lines was tested by qualitative RT-PCR.

### 5.2. Uptake of ^134^Cs

Plants were grown hydroponically in a growth chamber with constant growth conditions (25/15 °C day/night temperature, 16/8 light/dark cycle). Plants were grown for three weeks in 5.5L containers (18 plants per container) with aerated modified Murashige-Skoog media (0.2× strength, with NH_4_NO_3_ omitted) with the following nutrient composition (K^+^ replete medium with 4 mM K^+^): 3.76 mM KNO_3_, 0.25 mM KH_2_PO_4_, 0.3 mM MgSO_4_, 0.59 mM CaCl_2_, 20 µM H_3_BO_3_, 0.02 µM CoCl_2_, 0.02 µM CuSO_4_, 20 µM FeSO_4_ EDTA, 22.4 µM MnSO_4_, 0.21 µM Na_2_MoO_4_, 1 µM KI and 5.98 µM ZnSO_4_. NH_4_^+^ was omitted because it can block KT/HAK/KUP transporters or change their expression levels [11]. Cultivation media was replenished once a week. After three weeks of cultivation, fully developed plants were transferred into low K^+^ media (1 µM K^+^; other nutrients same as in K^+^ replete media) for one week. KH_2_PO_4_ and KNO_3_ were substituted by respective sodium salts in low K^+^ medium. pH was adjusted to 5.8 by NaOH in all cultivation media. Following low K^+^ treatment, plants were transferred into the incubation solution containing 1800 Bq.l^−1 134^CsCl and 0.5 mM CaCl_2_ for 24 h to monitor short-term ^134^Cs accumulation in plant tissues. After incubation, roots were washed with 0.5 mM CaCl_2_ solution for 3 min. Plants were divided into roots and shoots and plant parts were dried to a constant weight at 60 °C. Biomass of dried samples was weighed and activity of ^134^Cs was measured by HPGe gamma spectrometry in a well detector at National Radiation Protection Institute, Czech Republic.

### 5.3. Susceptibility to Cs^+^ Toxicity and K^+^ Shortage

To analyse plant sensitivity to caesium toxicity, we cultivated plants in vitro in agar-solidified media supplemented with various levels of ^133^Cs^+^ (stable isotope) and K^+^. The nutrient composition of germination medium (0.2 × Murashige-Skoog medium with omitted NH_4_NO_3_) was the same as in hydroponic cultivations (see above), and contained 4 mM K^+^. For potassium deficient media, KH_2_PO_4_ and KNO_3_ were substituted by respective sodium salts, and potassium levels (15 and 100 µM K^+^) were adjusted by addition of KCl. Caesium was added as ^133^CsCl to medium with 100 µM K^+^ to get 50, 100, and 200 µM Cs^+^ concentrations. Cultivation medium with 100 µM K^+^ and 200 µM Na^+^ (added as NaCl) was used to confirm the specificity of plant response to Cs^+^. Alternatively, low K^+^ medium with the exact salt composition of the low K^+^ media according to [28] was used to compare the growth of *kup7-1* and *kup7-2* mutant lines in low K^+^ conditions. This medium is based on 0.5 Murashige-Skoog medium and contains 100 µM K^+^. All media was supplemented with 1% *w*/*v* sucrose and solidified with 1% *w*/*v* agar (Plant agar; Duchefa, Netherlands). This agar contains 54 ± 2 mg/kg of residual potassium. This residual potassium was taken into account when potassium levels were adjusted. pH of the media was adjusted to 6.0 by NaOH.

Seeds were surface sterilized with 20% solution of commercial bleach with 0.1% triton for 15 min and washed with distilled water three times. Seeds were sown onto the solidified germination medium (see above) in the 100 × 100 mm sterile plates. The plates were then stored in 4 °C and darkness for three days to vernalise. After vernalisation, plates were placed into a cultivation room with constant growth conditions (22/18 °C day/night temperature, 16/8 h light/dark cycle). After five days, seedlings were randomly transferred onto solidified media with various K^+^ and Cs^+^ levels. Root system traits were determined 10 days after germination (DAG) and plant biomass on 16 DAG.

### 5.4. Measurements of Root System Traits and Plant Biomass

To monitor root system growth, plants on agar plates were scanned at high resolution (1200 dpi, 48-bit) and root system traits were measured by Root analyser plug-in in NIS Elements AR 3.22.05 software (Laboratory Imaging). To analyse plant growth and biomass production, roots and shoots were separated, and their fresh weights were measured. Plant parts were then dried to a constant weight at 60 °C to analyse dry weights and relative water contents.

### 5.5. Potassium Accumulation

Plants were grown in hydroponic containers (Araponics; Liege, Belgium) for 17 days supplied with aerated 4 mM K^+^ medium with NH_4_NO_3_ omitted (K^+^ replete medium with 4 mM K^+^; see above). The media was changed weekly. Then, plants were transferred to 15 µM K^+^ medium (media composition see above) for one week. Prior to harvest, plant roots were washed with 1 mM CaCl_2_ for 3 min. The biomass of the plants was determined. The dry mass was then homogenized by mixer mill (Retsch MM301) and subsequently decomposed by a solution of HNO_3_ and H_2_O_2_. Potassium content was measured by flame atomic absorption spectrometry on SpectrAA 280 FS (Varian; Palo Alto, CA, USA). In the second experiment, plants were grown for 16 days on solidified media supplied with 100 µM K^+^ and processed in the same way as plants from hydroponic culture.

### 5.6. Statistical Analyses

The statistical analysis was done with NCSS 9.0.15 software (Hintze, J. 2013. NCSS, LLC. Kaysville, UT, USA). The effects of treatments, genotypes, and their interaction were analysed with GLM ANOVA (Bonferroni Test). The differences between *kup7* plants and wild type controls in individual treatments were analysed with Aspin–Welch Unequal-variance T-test, *p* ≤ 0.05.

## Supplementary Materials

The following are available online at https://www.mdpi.com/2223-7747/9/11/1525/s1, Figure S1: Results of qualitative RT-PCR; Figure S2: Growth of plants in Cs^+^ uptake experiments; Figure S3: Growth of plants in K^+^ accumulation experiments; Figure S4: Comparison of growth in *kup7-1* and *kup7-2.*
